# The Jealousies and Bickerings in the Profession

**Published:** 1876-02

**Authors:** 


The Jealousies and Bickerings in the Profession.—We have just received the following letter from Dr. J. M. Johnson, of Atlanta, Georgia. We are pained to be forced to admit that all of which he complains is too true. We with pleasure insert the letter, with the hope that much good may result from it:

*Editors Atlanta Medical and Surgical Journal:*

I have a word to say to our brethren of the medical profession in Georgia; and not in Georgia only, but the medical profession elsewhere, for the same causes of reproach exist among doctors, go where you will. I mean jealousies, heartburnings, and evil speakings. From the tinker and cobbler up to the pulpit and bar, we have nothing like the amount of ill-concealed jealousy and distrust that is met with among the doctors; and it remains for the noblest of all the professions and occupations known among men to be riven by dissensions and bickerings.

Medical history, from the earliest period, discloses the fact that wrangling and personal conflict have been the rule. Galen, for teaching the theory of medicine, was obliged to flee from Rome to save his life. Contending colleges, and systems of education, and rivalries as unnecessary as they are undignified, have been to the science and art of medicine and surgery the marplot that has dimmed the glory and crippled and retarded the usefulness of both for centuries agone, and the end is not yet.

It is natural for men to disagree; they ought to do so upon speculative questions, until the truth can be reached; but why the enmities and railings so universal with us and so infrequent with other callings? All other professions, including the mechanics and trades people, have their festivals and hand-shakings; petty grievances and heart-aches are laid aside; they mingle freely together, and love each other better as they become better acquainted. But not so with the doctors. They hug their animosities to their hearts as though it was the one thing needful. They brush each other upon the sidewalk and at the hustings, looking daggers, or not looking at all. They stoop to trickery and low insinuation against a professional rival; maybe because he has qualities that carry him along on the current of popular favor, or if not this, from some other reason equally unworthy of a great and learned profession, which has given to the world more ideas and done more for humanity, science, and truth than all other professions put together; leaving out, of course, the plan of salvation, which was not wrought out and perfected by man, but by Him "who spake as never man spake." Is there to be no end to this state of things? Must this opprobrium, this crying shame, continue? Must envy, malice, hate—all these—continue to rankle and bear fruit, amid the temples which fame has crowned with immortality? The truths of its history surpass story or fable. Arabia, now but the winding sheet of her former self, gave ammonia and calomel, myrrh and opium to the world before Melchesideck met Abraham on the plains of Mamere and blessed him; before Agamemnon planned the seige of Troy, or Homer sang its fall; before eloquence was taught at Antioch, or statuary was made to speak the history of the ages past; before Socrates taught the immortality of the soul, or Cæsar crossed the Rubicon. It saw chemistry struggling in the womb of Alchemy; it kept time with Christianity through the dark ages; it heard the shackles fall when Magna Charter set an empire free, and to-day it brings the trophies of forty centuries and more to lay them at our feet, without money and without price.

Amid this glorious record, standing boldly forth and defying contradiction, we hear the discordant voices of contending biggots, the croakings of little minds, who "cry havock and let slip the dogs of war," not in the interest of humanity, religion, truth or science, but of turmoil and hate.

Great souls know nothing of such an atmosphere as this. They recognize the fact of their high calling; that the eyes of the world are upon them; that God has confided to their keeping the interests of an institution lying at the foundation of human society and human happiness; and it is their business to see that no unhallowed imprint is left upon its pure escutcheon. Their mission is to gather truths, the only offerings that science will accept from the hands of her devotees; lay these at her feet, and die; die "unwept, unhonored and unsung," it may be; but far beyond the wreck of dissolving worlds, where nothing but character can stand, a crown and sceptre await them.

I anticipate the inquiry, What is the remedy? Alas! there is none. Something may be done in the way of amelioration, but a cure is impossible so long as vain conceits revel where judgment and truth should reign supreme.

There are men who cannot look upon a rival, or admit a superior; who know and feel their inferiority, but who struggle to keep the world from knowing it, and to this end they whisper and wink and attempt, by malice in the dark, to make things even. The man who wastes his life in pleasure-hunting and prodigality, turns communist, and demands that his neighbor, who has been laborious and thrifty, shall divide with him. Such has been human nature from the beginning, and such it will be at the ending. But reforms are needed. It is Utopian to think of making men equal by education. The professional beggar will thank you for a nickel, while the college-bred beggar demands a suit of clothes, and money enough to take him to the centennial. By cheapening education you sometimes develope men into something who would otherwise be nothing, but generally you burden society with men who live and die drones, because they have been forced from their true base. Cheap medical education is a great factor in the evils we are deprecating. Many of our greatest men came from middle and even humble life. Nature had given them the stamina, and with such men obstacles are nothing. But the attempt to co-ordinate mankind by education, without giving them employment suited to their new ambition, and money besides, is utterly Utopian. Such men see nothing in society but its vices, and nothing in these but themselves. What has humanity and society gained?

J. M. Johnson.

*No.* 72 *Marietta street, Feb.* 8, 1876.

---

Dr. W. S. Kendrick.—In this number of the Journal the name of Dr. W. S. Kendrick appears upon the cover, as Associate Editor and Business Manager. For the past eighteen months he has been more or less intimately associated with the Journal, and by his energy and promptness has long since won the position now assigned him. In the future all letters connected with the business department of the Journal should be directed to Dr. W. S. Kendrick, Atlanta, Ga.

---

Errata.—The following errata occur in the article of Dr. Jno. M. Johnson, on "Opium Poisoning: First paragraph, fifth line, read had for have; third paragraph, fifth line, sodden for sudden; sixth paragraph, second line, transpose the words "sleep" and "stimulates."

